# Prognostic Effect of Bisphosphonate Exposure for Patients With Diagnosed Solid Cancer: A Systematic Review With Meta-Analysis of Observational Studies

**DOI:** 10.3389/fonc.2018.00495

**Published:** 2018-10-29

**Authors:** Dan-Ting Wen, Zheng Xu, Mei-Ling Xuan, Guo-Rong Liang, Wei-Ling Zheng, Xue-Fang Liang, Jing Xiao, Xiao-Yun Wang

**Affiliations:** ^1^Department of Gynecology, Second Affiliated Hospital of Guangzhou University of Chinese Medicine, Guangzhou, China; ^2^Postdoctoral Research Station, Guangzhou University of Chinese Medicine, Guangzhou, China; ^3^Department of Gynecology, Guangdong Provincial Hospital of Traditional Chinese Medicine, Guangzhou, China; ^4^General Office of Multiple Functional Chinese Medications, Bao'an TCM Hospital Group, Shenzhen, China

**Keywords:** bisphosphonate, solid cancer, survival, prognosis, cohort study, nested case-control study

## Abstract

**Background:** Bisphosphonates are widely prescribed for the prevention and treatment of osteoporosis. Recent epidemiological studies indicate that people with bisphosphonate use may have lower cancer risk and have improved survival. The aim of this study is to determine the association between bisphosphonate use and survival outcomes in solid cancer patients using systematic review and meta-analysis.

**Methods:** A systematic literature search was performed using the PubMed, Embase, and Cochrane databases. Original articles published until April, 2018 were selected. The survival outcome measures assessed included overall survival (OS), cancer-specific survival (CSS) and recurrence-free survival (RFS). Pooled hazard ratio (HR) and their 95% confidence interval (95% CI) were derived using a random-effects model.

**Results:** Out of 9,742 retrieved citations, six cohort studies and two nested case-control studies satisfying the inclusion criteria were included for analyses. Bisphosphonate use was significantly associated with improved OS (HR 0.84, 95% CI 0.76–0.93), CSS (HR 0.73, 95% CI 0.58–0.90) and RFS (HR 0.72, 95% CI 0.53–0.96). The results of subgroup analyses stratified by major study characteristics were generally consistent with the main findings. For individual cancer type, we found that bisphosphonate use was significantly associated with longer OS for patients with gastroesophageal cancer (HR 0.62, 95% CI 0.40–0.98), as well as longer CSS for patients with breast cancer (HR 0.73, 95% CI 0.55–0.95).

**Conclusions:** Current evidence indicates that bisphosphonate use is significantly associated with improved survival for patients with solid cancer. However, the prognostic effects in specific solid tumors remains to be confirmed by further large prospective cohort studies.

## Introduction

Cancer is the second major cause of morbidity and mortality worldwide behind cardiovascular diseases, with an estimated over 8.7 million deaths in 2015 (1). Moreover, the number of incident cases has been rising dramatically and patients tend to be younger (2, 3). Although a large number of cancers could currently be diagnosed at an early stage, survival rates have not improved largely. Surgery remains the mainstay of treatment in clinically localized disease for solid tumors, but despite this option, 20–30% of those with localized disease could experience progression or recurrence postoperatively, and the most of those patients would die of the disease progression (4). However, no definite therapies have been identified to reduce the risk of recurrence, progression or death from cancer after surgical intervention. Over the past few years, tremendous advancements in personalized medicine and novel treatment options like targeted therapy and immunotherapy have raised hope of greatly prolonging cancer patient survival (2, 5).

Bisphosphonates are commonly-prescribed medications widely prescribed for the prevention and treatment of osteoporosis. They are generally well-tolerated with the most common side effects being renal toxicity, gastrointestinal reactions, osteonecrosis of the jaw, atypical fractures, atrial fibrillation, musculoskeletal complaints, and esophageal cancer (6–8). Currently, bisphosphonates have gained popularity within the oncology prevention and treatment fields, based on studies indicating reduced cancer risk and patient survival (9–11). Findings from epidemiological studies have prompted clinical trials evaluating bisphosphonates combined with other medications or as a single agent in the neoadjuvant and adjuvant setting in patients with solid cancer (12). Though the antineoplastic effects of bisphosphonates are still unclear, preclinical studies have demonstrated that they can affect tumor cell proliferation, growth-factor release, cancer-cell adhesion, invasion, and angiogenesis which might be enhanced through co-administration with chemotherapy agents, biological agents, or both (13–15). Moreover, bisphosphonates are also reported to affect tumor microenvironment degradation proteins and induce apoptosis (16, 17). They have also been indicated to exert antitumor effects of modulating cancer progression and metastasis through the mevalonate pathway (18).

Several observational studies have found potential associations between bisphosphonate exposure and cancer risk/survival including gastrointestinal cancer, colorectal cancer, endometrial and ovarian cancer, and these studies reported inconsistent findings (11, 19–21). Thus, we aimed to conduct a meta-analysis to examine whether use of bisphosphonates was associated with differential survival outcomes in patients diagnosed with solid cancer.

## Materials and methods

### Search strategy

This meta-analysis was performed in accordance with the PRISMA guidelines (22) (Table S4). We conducted a computer-assisted search with the databases including PubMed, Cochrane Library, and Embase to identify relevant published studies on the prognostic effect of bisphosphonate use on the survival of solid cancers. We searched the databases from inception through March, 2018 with the following keywords: “diphosphonates or bisphosphonates” and “neoplasm^*^ or cancer or tumor or tumor or carcinoma or malignanc^*^” and “survival or mortality or death or prognos^*^ or outcome” (Tables S1–S3). Manual searches of reference lists from eligible studies were performed to identify potential missed studies during the computer-assisted search. We also conducted additional manual searches of some major journals on clinical oncology, such as “Lancet Oncology,” “Journal of Clinical Oncology,” “Annals of Oncology” and “Journal of The National Cancer Institute,” and abstract from the American Society of Clinical Oncology (ASCO) meeting. Moreover, we also consulted expert oncologists to identify additional references or unpublished literature.

### Study selection and inclusion criteria

Two authors (DW and ZX) reviewed all titles or abstracts of citations identified by the database search to retrieve potentially relevant articles. The full-text for each potentially relevant article was then reviewed by each of the two authors. The articles were cross checked by two independent authors for possible inclusion and disagreements were resolved through discussion or by consensus with a senior author (XW or JX).

Observational studies were satisfied the inclusion criteria and thus included for analysis if they (i) included patients clinically or pathologically diagnosed with solid cancer, (ii) evaluated and clearly defined exposure to any bisphosphonates, (iii) reported survival outcomes in patients with solid cancer, and (iv) reported hazard ratios (HRs) or relative risks (RRs) and their corresponding 95% CIs or provided relevant data for their calculation. If several publications shared the same cohort, we used the article with the most informative one.

### Data abstraction

Two authors (DW and ZX) independently conducted data abstraction using a standardized form. The following data were summarized from each study: first author, publication year, tumor type, study design, study country, sex, sample size, follow-up duration, HR and 95% confidence intervals (CIs) with fully adjustment for confounding variables. Conflicts were resolved by discussion or by a senior author. One author (XW) assessed the quality of each study by Ottawa–Newcastle scale (NOS). After the resolution of discrepancies, a final NOS score for observational studies was obtained based on selection of involved population, comparability of study groups, and adequacy of outcome assessment (23).

### Outcomes assessed

The primary outcome measure was OS which defined as the time from the enrollment to death from any cause. The secondary outcome measures included CSS, defined as the time from the enrollment to death from a specific cancer type, and RFS, defined as the time from the enrollment to the first recorded disease recurrence or death from any cause, which would occurred first.

### Statistical analysis

Stata® version 12.0 (Stata Corp LP, College Station, Texas, USA) was applied to perform all statistical analyses. We used the DerSimonian and Laird random-effects model to calculate summarized HR and 95% CI (24). Summary estimates reported in studies adjusted for most confounding variables were used for analysis. Inter-study heterogeneity was tested by using Cochrane's *Q* test, defined as statistically significant if *p*-value was < 0.10 or I^2^ statistic if an I^2^ statistic more than 50% representing significant heterogeneity (25). Random-effects models were used to meta-analyze each of the primary and secondary outcome measure. Subgroup analyses were also applied to examine the potential sources of heterogeneity, such as cancer type. Publication bias was assessed by visual inspection funnel plot asymmetry along with Begg's rank correlation test and Egger's linear regression test, with a *P*-value < 0.1 as an indication of publication bias (26, 27).

Sensitivity analyses were performed to further investigate the sources of inter-study heterogeneity in terms of some major study characteristics for OS and CSS subset, which was also used to examine the robustness of the findings. The influence of individual study on the pooled estimates was conducted by omitting one study at a time and recalculating the others. Furthermore, subgroup differences were conducted using meta-regression analysis when necessary. A *P*-value of < 0.05 was considered statistically significant.

## Results

### Literature search

**Figure 1:**
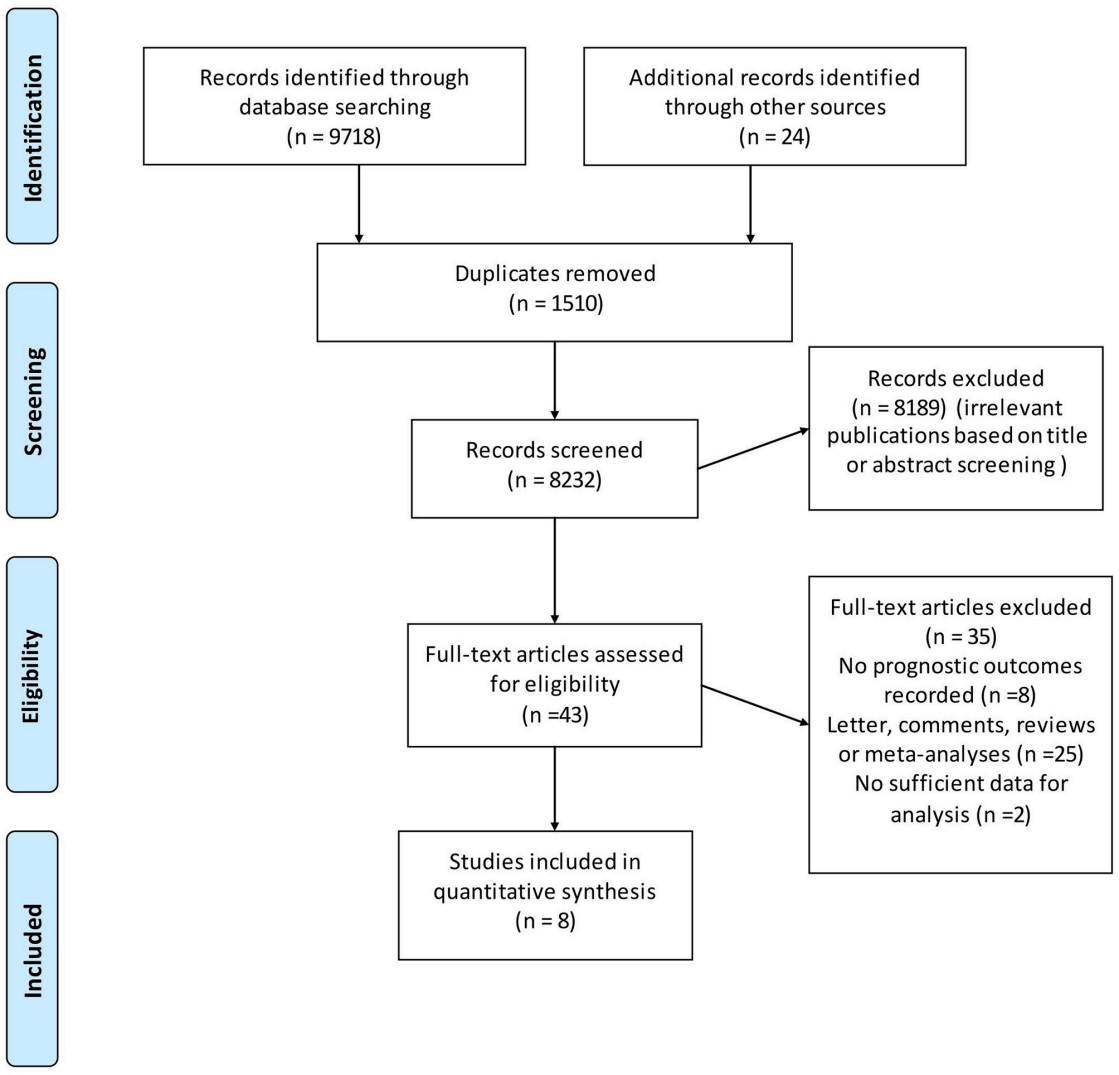
Flow chart for the process of identifying studies included in and excluded from the systematic review.

The database search yielded a total of 9,742 unique citations published through March, 2018. After title or abstract review, 43 records were potentially appropriate, which were further reviewed through full-text reading, and an additional 35 studies were excluded due to various reasons. Finally, eight studies with a total of 241,634 individuals met the inclusion criteria (Figure 1) for inclusion in the meta-analysis (19, 28–34).

### Study characteristics

The characteristics of the included studies are shown in Table 1. These studies were published from 2012 to 2018. Six of these studies were cohort studies (19, 28–30, 33, 34), and the remaining two were nested case-control studies (31, 32). Three studies were each from Europe and USA/Canada, respectively, and two studies represented the Asian population. Four studies investigated survival benefits of bisphosphonates in breast cancer patients (28–31), three in colorectal cancer patients (19, 32, 33) and one in gastroesophageal cancer patients (34). The median sample size of the included studies was 10,786 (range 1,706–153,030). The quality of the included studies was generally high with five of eight stars and three of nine stars.

**Table 1:** Major baseline features of the included studies.

**References**	**Study design**	**Tumor location**	**Study setting**	**Country**	**Sex**	**Exposure ascertment**	**Sample size**	**Follow-up**	**NOS**
Korde et al. (28)	Population-based cohort study	Early stage invasive breast cancer	Multiple center	USA	Female	Medical record, interview, and cancer registry data	1,813	Median 11.8 years	8
Kwan et al. (29)	Hospital-based cohort study	Breast cancer	Multiple center	USA	Female	Electronic health records and cancer registries	16,781	Median 6.4 years	8
Hicks et al. (19)	Prospective population-based cohort study	Colorectal cancer	Multiple center	UK	Male/female	The UK Clinical Practice Research Datalink (CPRD)	4,791	Mean 3.3 years	9
Kremer et al. (30)	Community-based cohort study	Breast cancer	Multiple center	Canada	Female	Prescription recorded database	21,664	Median 5 years	9
Rennert et al. (31)	Population-based nested case-control study	Breast cancer	Multiple center	Israel	Female	Computerized prescription records	3,731	Mean 5.83 years	8
Rennert et al. (32) (Breast cohort)	Population-based nested case-control study	Breast cancer	Multiple center	Israel	Female	Pharmacy records	1,706	Mean 5.8 years	8
Pazianas et al. (33) (Colon cohort)	Population-based national register-based cohort study,	Colon cancer	Multiple center	Denmark	Male/female	Medical record	38,118	Mean 4.9 years	8
Abrahamsen et al. (34)	Population-based national register-based cohort study,	Esophageal and Gastric Cancer	Multiple center	USA, Danmark	Male/female	Record of all hospitalizations and outpatient appointments	153,030	Mean 3.5 years	9

*CI, confidence interval; het, heterogeneity; HR, hazard ratio*.

### Relationship between bisphosphonate use and solid cancer survival

**Figure 2:**
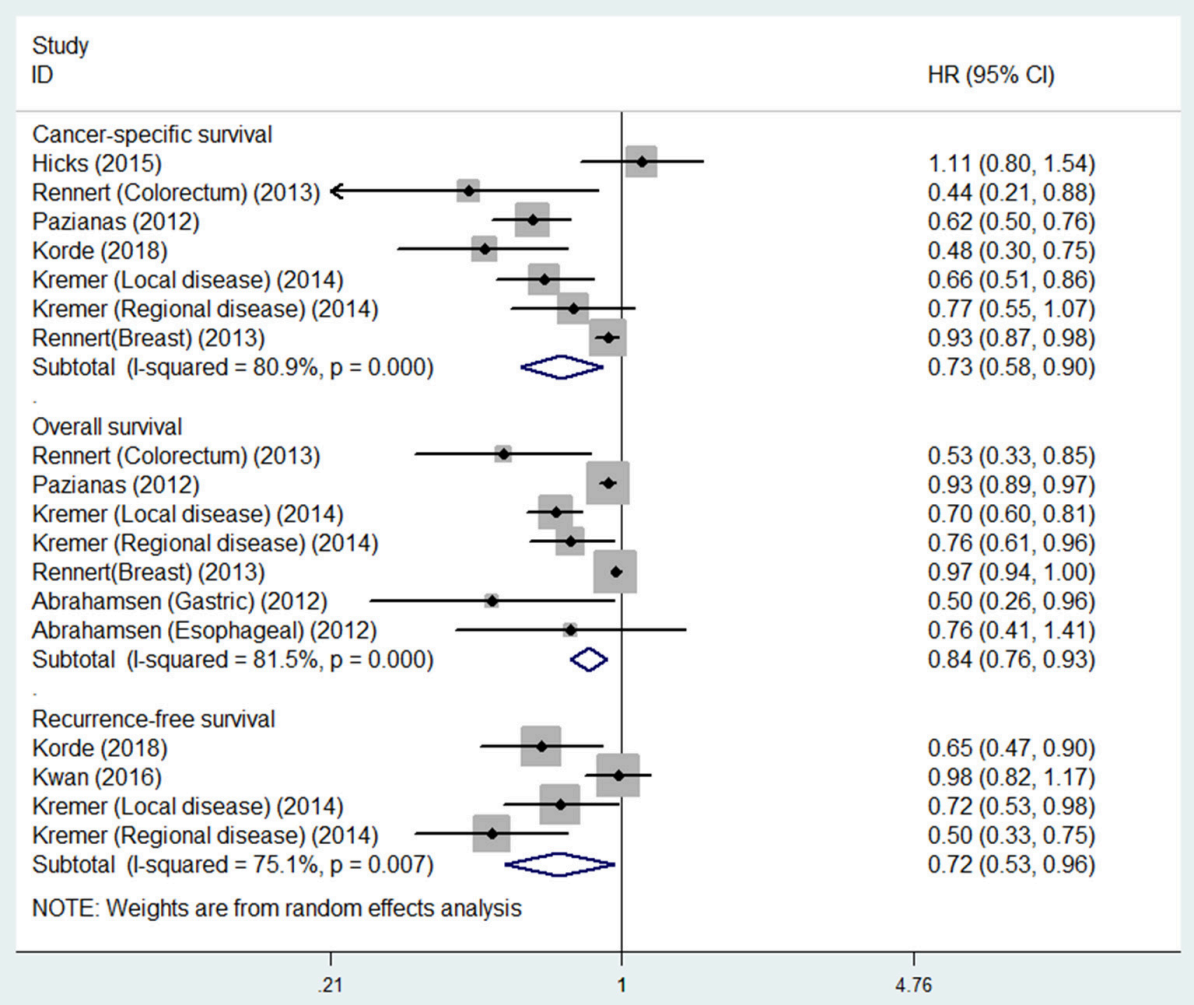
Summary estimates and 95% CIs for overall survival, cancer-specific survival and recurrence-free survival for associations between bisphosphonate use and survival of patients with solid tumors. Weights are from random effects analysis. CI, confidence interval; HR, hazard ratio; W (random), Weights (random effects model).

Five studies were involved in the analysis of bisphosphonate use and OS. Figure 2 showed that bisphosphonate use was associated with prolonged OS (HR 0.84, 95% CI 0.76–0.93). We noted that there was evidence of heterogeneity (I^2^ = 81.4%).

Six studies were involved in the analysis of bisphosphonate use and CSS. The result showed that bisphosphonate use was associated with prolonged CSS (HR 0.73, 95% CI 0.58–0.90). We noted that there was evidence of heterogeneity (I^2^ = 80.9%).

Three studies were involved in the analysis of bisphosphonate use and RFS. The result showed that bisphosphonate use was associated with prolonged RFS (HR 0.72, 95% CI 0.53–0.96). We noted that there was evidence of heterogeneity (I^2^ = 75.1%; Table 2).

**Table 2:** Meta-analysis of associations between bisphosphonate use and survival of patients with solid tumors.

**Variable**	**No of studies**	**HR (95% CI)**	***P*-value**	**I^2^, *P*_het_**
Overall survival	5	0.84 (0.76–0.93)	< 0.001	81.5, < 0.001
Cancer-specific survival	6	0.73 (0.58–0.90)	< 0.001	80.9, < 0.001
Recurrence-free survival	3	0.72 (0.53–0.96)	< 0.001	75.1, < 0.001
**TUMOR LOCATION**				
**Overall survival**				
Colorectal cancer	2	0.74 (0.43–1.27)	0.02	81.4, < 0.001
Breast cancer	2	0.81 (0.63–1.04)	< 0.001	90.7, < 0.001
Gastroesophageal cancer	1	0.62 (0.40–0.98)	< 0.001	81.5, < 0.001
**Cancer-specific survival**				
Colorectal cancer	3	0.71 (0.44–1.15)	0.005	80.9, < 0.001
Breast cancer	3	0.73 (0.55–0.95)	0.002	79.4, < 0.001

*CI, confidence interval; het, heterogeneity; HR, hazard ratio*.

The results of subgroup analyses for the associations between bisphosphonate use and OS or CSS were presented in Tables 3A, 3B. Generally, the findings of most of the subgroups were statistically significant, favoring that bisphosphonate use was significantly associated with improved survival for solid cancer patients, which was consistent with the main results. Furthermore, we noted that heterogeneity for the majority of the subgroups significantly reduced, indicating the sources of heterogeneity might originate from difference in geographical location, study quality, study sample size, study design, gender, tumor stage, source of exposure, statistical method (Table 3).

**Table 3A:** Subgroup analyzes for the associations between bisphosphonate use and overall survival of patients with solid tumors.

**Variables**	**No. of study**	**HR (95%CI)**	**I^2^ (%)**	P^*^	P^**^
Geographical location				< 0.001
Europe	3	0.79 (0.57–1.12)	48.1	0.146
America	2	0.72 (0.63–0.81)	0	0.553
Asia	2	0.75 (0.42–1.35)	84	0.012
Study quality				< 0.001
Low risk	3	0.94 (0.88–1.01)	76.3	0.015
High risk	4	0.71 (0.63–0.80)	0	0.674
Number of cases				
< 10,000	2	0.75 (0.42–1.35)	84	0.012	0.006
≥10,000	5	0.77 (0.63–0.94)	78.4	0.001
Type of cohort				0.011
Retrospective	4	0.71 (0.48–1.05)	72.1	0.013
Prospective	3	0.80 (0.65–0.99)	86.7	0.001
Gender				0.41
Female	6	0.74 (0.59–0.92)	84.2	< 0.001
Both gender	1	0.93 (0.89–0.97)	N/A	N/A
Tumor stage				< 0.001
I–IV	5	0.92 (0.85–1.00)	68.6	0.013
I–III	2	0.72 (0.63–0.81)	0	0.553
Source of exposure				N/A
Prescription database	7	0.84 (0.76–0.93)	81.4	< 0.001
Medical records	0	N/A	N/A	N/A
Statistical analysis				0.07
Time-dependent	5	0.85 (0.77–0.94)	85.8	< 0.001
Non time-dependent	2	0.62 (0.40–0.98)	0	0.361
Adjustment for major confounders				0.303
Yes (Age, stage and grade)	4	0.76 (0.60–0.97)	89	< 0.001
No (Age, stage and grade)	3	0.79 (0.57–1.12)	48.1	0.146

*CI, confidence interval; HR, hazard ratio; N/A, not available*;

* *P-value for heterogeneity within each subgroup*;

** *P-value for heterogeneity between subgroups in meta-regression analysis*.

**Table 3B:** Subgroup analyses for the associations between bisphosphonate use and cancer-specific survival of patients with solid tumors.

**Variables**	**No. of study**	**HR (95%CI)**	**I^2^ (%)**	P^*^	P^**^
Geographical location					< 0.001
Europe	2	0.82 (0.46–1.45)	88.4	0.003
America	3	0.65 (0.52–0.82)	25.3	0.262
Asia	2	0.70 (0.34–1.43)	76.0	0.041
Study quality					0.255
Low risk	4	0.64 (0.45–0.92)	87.5	< 0.001
High risk	3	0.82 (0.60–1.11)	66.5	0.050
Number of cases					< 0.001
< 10,000	4	0.76 (0.54–1.07)	77.3	0.004
≥10,000	3	0.66 (0.57–0.76)	0	0.558
Type of cohort					< 0.001
Retrospective	2	0.70 (0.34–1.43)	76.0	0.041
Prospective	5	0.71 (0.56–0.89)	66.7	0.017
Gender					0.037
Female	5	0.69 (0.52–0.91)	78.2	0.001
Both gender	2	0.82 (0.46–1.45)	88.4	0.003
Tumor stage					0.002
I–IV	4	0.79 (0.59–1.06)	83.9	< 0.001
I–III	3	0.65 (0.52–0.82)	25.3	0.262
Source of exposure					0.009
Prescription database	6	0.77 (0.62–0.95)	79.7	< 0.001
Medical records	1	0.48 (0.30–0.76)	N/A	N/A
Statistical analysis					N/A
Time-dependent	7	0.73 (0.58–0.90)	80.9	< 0.001
Non time-dependent	0	N/A	N/A	N/A
Adjustment for major confounders					0.001
Yes (Age, stage and grade)	6	0.75 (0.60–0.95)	75.0	0.001
No (Age, stage and grade)	1	0.62 (0.50–0.76)	N/A	N/A

*CI, confidence interval; HR, hazard ratio; N/A, not available*;

* *P-value for heterogeneity within each subgroup*;

** *P-value for heterogeneity between subgroups in meta-regression analysis*.

### Bisphosphonate use and survival in different cancer types

**Figure 3:**
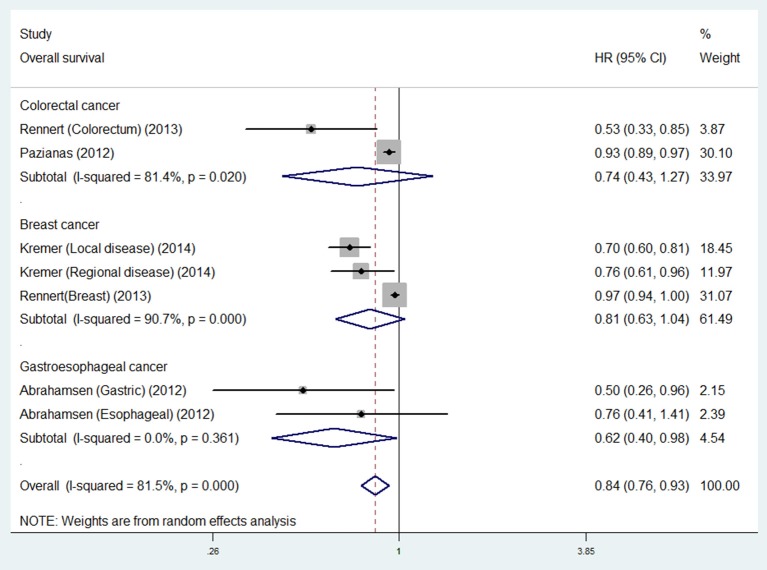
Summary estimates and 95% CIs for overall survival associations between bisphosphonate use and survival of patients with solid tumors according to tumor location. Weights are from random effects analysis. CI, confidence interval; HR, hazard ratio; W (random), Weights (random effects model).

**Figure 4:**
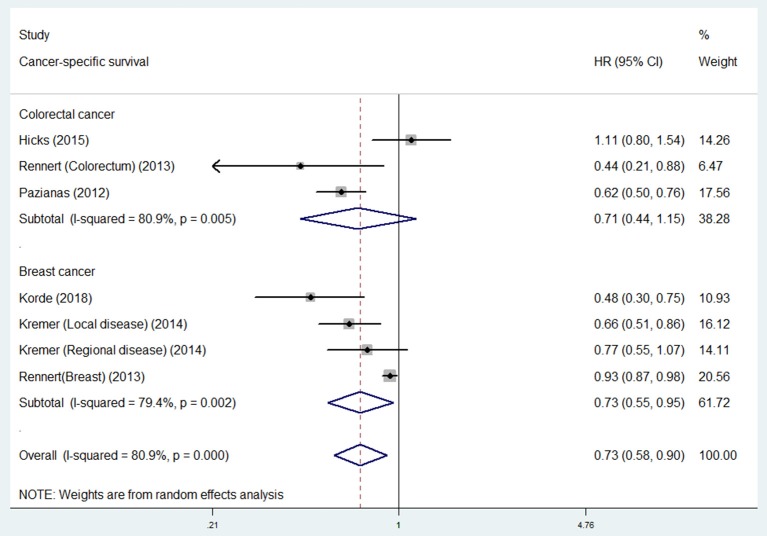
Summary estimates and 95% CIs for cancer-specific survival of associations between bisphosphonate use and survival of patients with solid tumors according to tumor location. Weights are from random effects analysis. CI, confidence interval; HR, hazard ratio; W (random), Weights (random effects model).

For individual cancer type, we found that bisphosphonate use was significantly associated with longer OS for patients with gastroesophageal cancer (HR 0.62, 95% CI 0.40–0.98), but not for patients with breast (HR 0.74, 95% CI 0.43–1.27) or colorectal cancer (HR 0.81, 95% CI 0.63–1.04; Figure 3). Besides, bisphosphonate use was significantly associated with longer CSS for patients with breast cancer (HR 0.73, 95% CI 0.55–0.95), but not for patients with colorectal cancer (HR 0.71, 95% CI 0.44–1.15; Figure 4).

### Publication bias and sensitivity analyses

As there were limited number of studies (< 10) included in each study outcome subset, we did not assess publication bias. Sensitivity analyses by omitting one study at a time and recalculating the summary HRs for the remaining studies yielded consistent results for each outcome of OS, CSS and RFS subset (Figures S1–S3).

## Discussion

In this meta-analysis of eight studies investigating the prognostic effect of bisphosphonate use on the survival of patients with solid cancer, we found that bisphosphonate use was associated with improved survival for patients with solid cancer in terms of OS, CSS and RFS. However, we did not find improved OS or CSS in all type of solid cancers. We noted considerable inter-study heterogeneity that could be explained by study design, tumor stage or sample size.

This meta-analysis is the first one to provide evidence for substantial prognostic role of bisphosphonate use in patients with solid cancer. Although several previous studies showed no prognostic association of bisphosphonate use in solid cancers (19, 29, 34), there was limited statistical power due to the small sample sizes in single study. These inconsistent results of the included studies might attribute to several factors. Firstly, the included studies had various sample sizes and we know that studies with smaller sample size could result in inconsistent risk estimates of effect sizes, which was prone to publication bias. Secondly, different study designs were applied to the included studies; some studies which conducted in single center used a convenience sample, mainly with huge selection bias, while others used samples from population-based samples or multiple institutions or in epidemiologic study settings. Thirdly, the basic disease characteristics, such as disease stage, tumor location, adjuvant treatment, follow-up duration, bisphosphonate type, dose and duration vary among studies, which might cause potential heterogeneity in study findings. In this study, we thoroughly conducted subgroup analyses according to tumor location and other potential major study characteristics for outcomes of OS and CSS and the findings were generally consistent and we initially considered that inter-study heterogeneity could attribute to these characteristics. Moreover, we found that there was little inter-study heterogeneity in stage I-III patients for OS and CSS subsets, and also in large studies (sample size ≥ 10,000; Tables 3A, 3B). Due to limited studies, further large prospective studies should be conducted in these subsets in the future. Fourthly, some more important molecular pathological data including KRAS, BRAF, PIK3CA, HER-2, ER, PR, and PD-L1 status, microsatellite instability status, CpG island methylator phenotype, microRNA expression, and other influential prognostic factors, which had been identified to be associated with specific cancer patient survival (35–42), could not be analyzed separatedly due to unavailability of these data in the included studies. Fifthly, due to the nature of observational study, we could not conclude the casual relationship between bisphosphonate use and reduced cancer mortality. Finally, we did not include gray literature or other unpublished literature, having the potentials to lead to publication bias.

Despite the above limitations, the present study also has several strengths. Firstly, we developed a set of comprehensive database search strategy for search without language limits, which could reduce the risk of missing studies to a great deal. Secondly, this is the largest meta-analysis with more than 153,000 participants involved which provided the most powerful evidence regarding this subject ever to date. Thirdly, at least two authors independently did the literature search, study selection and study quality assessment and the results were cross-checked by a senior author, which guaranteed the objective assessment of the systematic review.

Studies have indicated that several major side effects or adverse events are closely related to bisphosphonate use. It seems that people with bisphosphonate use have a higher risk of developing acute-phase reactions (fever, myalgias, lymphopenia, etc.), hypocalcemia, iritis, renal toxicity and osteonecrosis of the jaw (43–47). Despite all these side-effects, the major clinical implications of this study lies in that bisphosphonate use is significantly associated with improved survival for patients with solid cancer. Osteoporosis is a common status in a substantial number of patients diagnosed with malignancy who take bisphosphonates and bisphosphonate exposure is strongly correlated with decreased mortality and improved progression, even after adjustment for some clinical variables. Future research should be focused on large prospective studies or individual patient data meta-analyses to minimize the confounders in specific malignancies; moreover, we also consider more in that current evidence lacks evidence regarding drug dose and duration response of bisphosphonate use.

## Author contributions

X-YW and JX study concept and design. D-TW, ZX, M-LX, G-RL, W-LZ, and X-FL acquisition of data. D-TW, X-YW, and JX analysis and interpretation of data. D-TW and X-YW drafting of the manuscript. All authors critical revision of the manuscript for important intellectual content. X-YW and JX study supervision.

### Conflict of interest statement

The authors declare that the research was conducted in the absence of any commercial or financial relationships that could be construed as a potential conflict of interest.
