# Supplementary material for: Prognostic Effect of Bisphosphonate Exposure for Patients With Diagnosed Solid Cancer: A Systematic Review With Meta-Analysis of Observational Studies
Source: Front Oncol. 2018 Oct 29;8:495. doi: 10.3389/fonc.2018.00495 (PMC6215818; doi:10.3389/fonc.2018.00495)
Supplement: Supplementary file 1 [file Table_1.DOCX]

**Table S1. Pubmed search strategy**

| 1. "Diphosphonates"[Mesh] |
| --- |
| 1. Bisphosphonate*[Title/Abstract] |
| 1. 1 OR 2 |
| 1. "Neoplasms"[Mesh] |
| 1. (cancer* OR tumor* OR tumour* OR carcinom* OR neoplas* OR malignan* OR adenocarcinoma) [Title/Abstract] |
| 1. 4 OR 5 |
| 1. "Survival"[Mesh] OR "Mortality"[Mesh] |
| 1. "Prognosis"[Mesh] |
| 1. (prognos* OR survival OR recurren* OR mortality OR predict* OR outcome* OR death)[Title/Abstract]) |
| 1. 7 OR 8 OR 9 |
| 1. 3 AND 6 AND 10 |
