# Supplementary material for: Prognostic Effect of Bisphosphonate Exposure for Patients With Diagnosed Solid Cancer: A Systematic Review With Meta-Analysis of Observational Studies
Source: Front Oncol. 2018 Oct 29;8:495. doi: 10.3389/fonc.2018.00495 (PMC6215818; doi:10.3389/fonc.2018.00495)
Supplement: Supplementary file 2 [file Table_2.DOCX]

**Table S2. Search strategy for Embase**

| 1. 'bisphosphonic acid derivative'/exp |
| --- |
| 1. ' bisphosphonates:ab,ti |
| 1. 1 OR 2 |
| 1. 'colorectal cancer'/exp |
| 1. 'colon cancer'/exp |
| 1. 'rectum cancer'/exp |
| 1. ((colorect* OR colon* OR rectum OR rectal) and (cancer* OR tumor* OR tumour* OR carcinom* OR neoplas* OR adenocarcinoma* OR malignan*)):ab,ti |
| 1. 4 OR 5 OR 6 OR 7 |
| 1. 3 AND 8 |
