# Supplementary material for: Prognostic Effect of Bisphosphonate Exposure for Patients With Diagnosed Solid Cancer: A Systematic Review With Meta-Analysis of Observational Studies
Source: Front Oncol. 2018 Oct 29;8:495. doi: 10.3389/fonc.2018.00495 (PMC6215818; doi:10.3389/fonc.2018.00495)
Supplement: Supplementary file 3 [file Table_3.DOCX]

**Table S3. Search strategy for the Cochrane Library Central Register of Controlled Trials**

| 1. MeSH descriptor: [Diphosphonates] explode all trees |
| --- |
| 1. Bisphosphonates:ti,ab,kw (Word variations have been searched) |
| 1. 1 OR 2 |
| 1. MeSH descriptor: [Colorectal Neoplasms] explode all trees |
| 1. MeSH descriptor: [Colonic Neoplasms] explode all trees |
| 1. MeSH descriptor: [Rectal Neoplasms] explode all trees |
| 1. (colorect* or colon* or rectum or rectal) and (cancer* or tumor* or tumour* or carcinom* or neoplas* or adenocarcinoma* or malignan*):ti,ab,kw |
| 1. 4 OR 5 OR 6 OR 7 |
| 1. 3 AND 8 |
