# Supplementary figures and images for: Prognostic Effect of Bisphosphonate Exposure for Patients With Diagnosed Solid Cancer: A Systematic Review With Meta-Analysis of Observational Studies
Source: Front Oncol. 2018 Oct 29;8:495. doi: 10.3389/fonc.2018.00495 (PMC6215818; doi:10.3389/fonc.2018.00495)

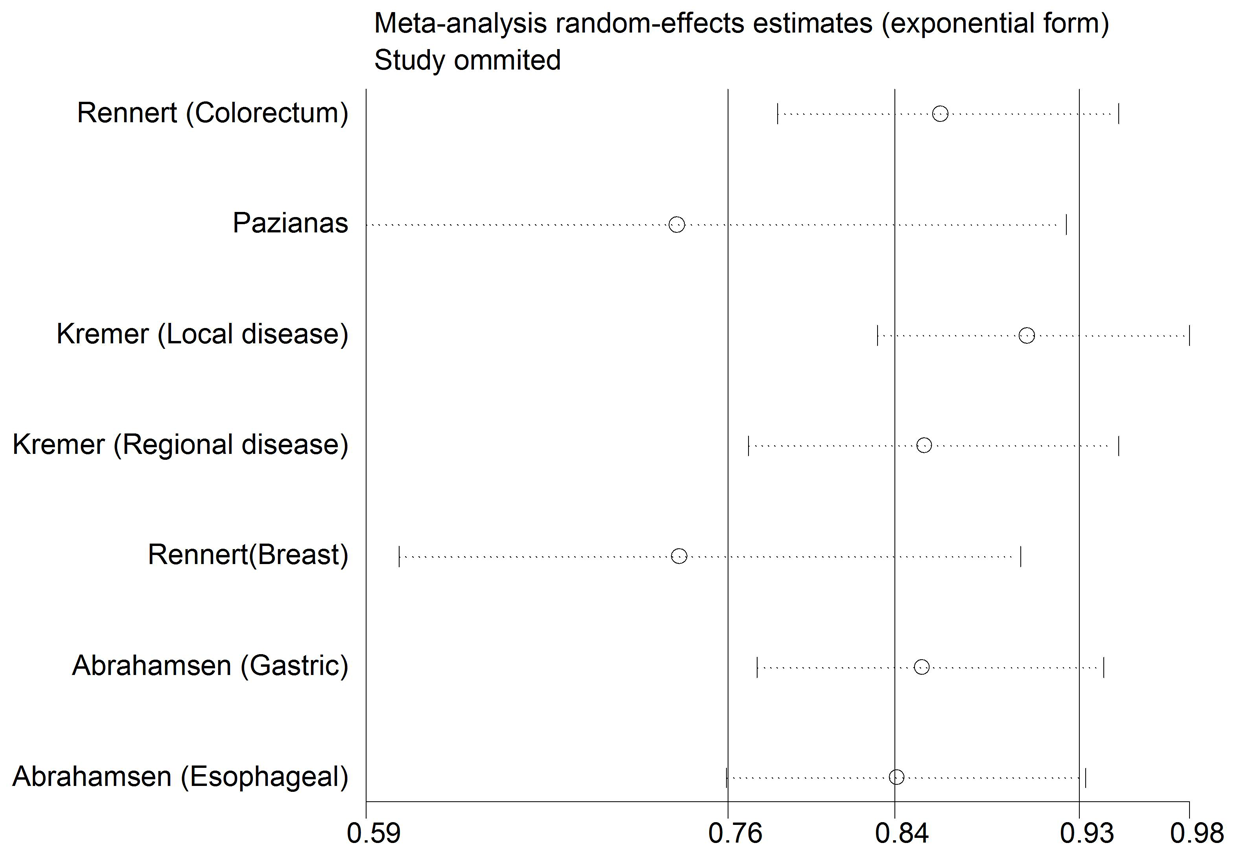

Supplement: Supplementary file 5 [file Image_1.TIF]

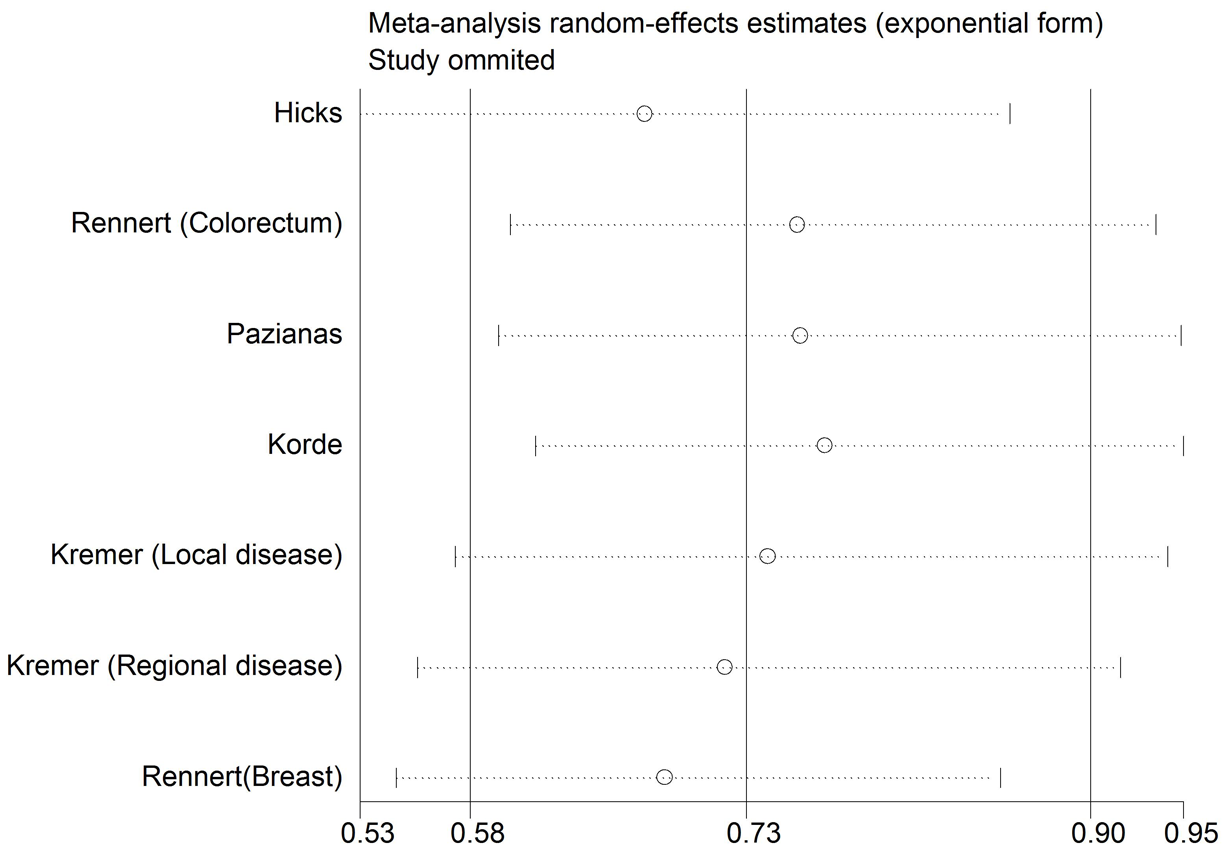

Supplement: Supplementary file 6 [file Image_2.TIF]

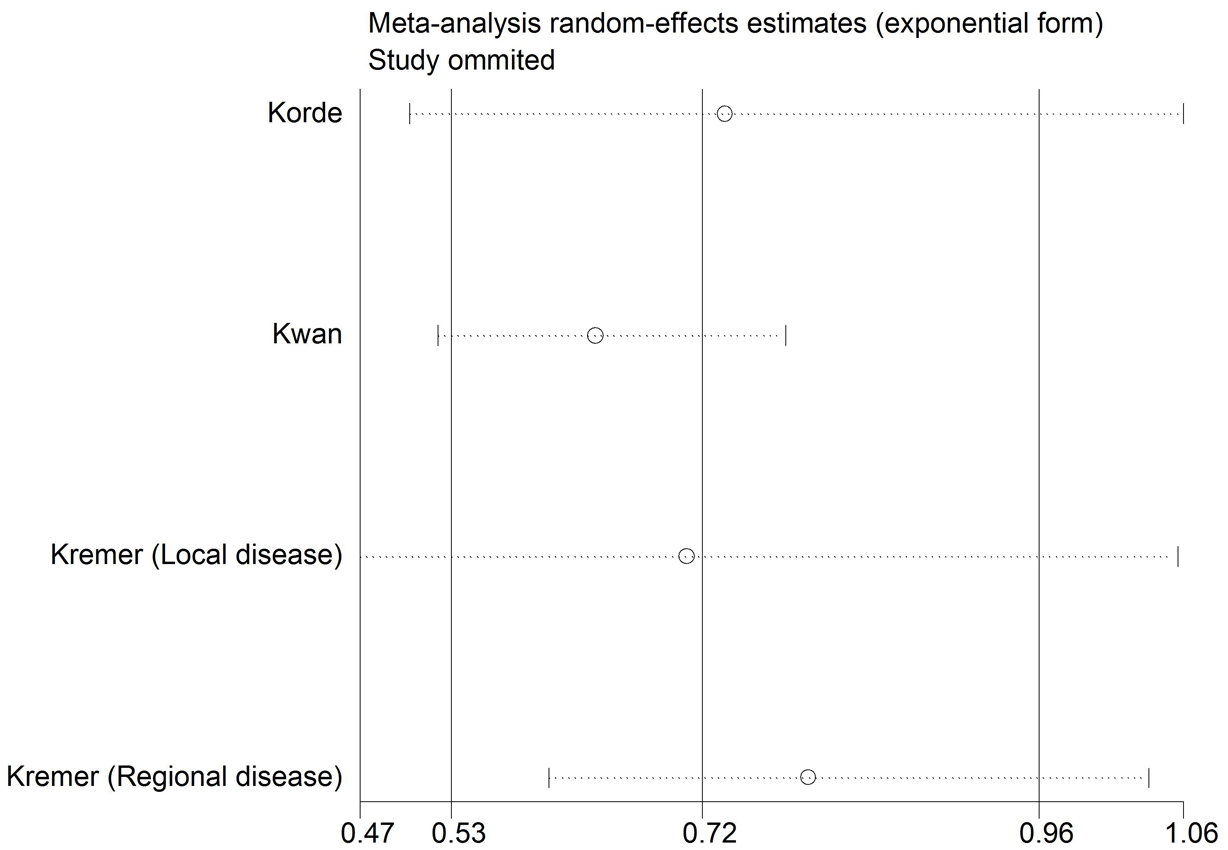

Supplement: Supplementary file 7 [file Image_3.TIF]
